# Engineering an aldehyde dehydrogenase toward its substrates, 3-hydroxypropanal and NAD^+^, for enhancing the production of 3-hydroxypropionic acid

**DOI:** 10.1038/s41598-017-15400-x

**Published:** 2017-12-07

**Authors:** Ye Seop Park, Un Jong Choi, Nguyen Hoai Nam, Sang Jin Choi, Abdul Nasir, Sun-Gu Lee, Kyung Jin Kim, Gyoo Yeol Jung, Sangdun Choi, Jeung Yeop Shim, Sunghoon Park, Tae Hyeon Yoo

**Affiliations:** 1 Department of Molecular Science and Technology, Ajou University, 206 World cup-ro, Yeongtong-gu, Suwon, 16499 Korea; 2 School of Energy and Chemical Engineering, Ulsan National Institute of Science and Technology (UNIST), Ulsan, 44919 Korea; 3 Department of Chemical and Biomolecular Engineering, Pusan National University, Pusan, 46241 Korea; 4 School of Life Sciences, Kyungpook National University, Daegu, 41566 Korea; 5 Department of Chemical Engineering, Pohang University of Science and Technology, Pohang, 37673 Korea; 6 Bio R&D Center, Noroo Holdings Co., Ltd, Suwon, 16229 Korea

## Abstract

3-Hydroxypropionic acid (3-HP) can be produced via the biological route involving two enzymatic reactions: dehydration of glycerol to 3-hydroxypropanal (3-HPA) and then oxidation to 3-HP. However, commercial production of 3-HP using recombinant microorganisms has been hampered with several problems, some of which are associated with the toxicity of 3-HPA and the efficiency of NAD^+^ regeneration. We engineered α-ketoglutaric semialdehyde dehydrogenase (KGSADH) from *Azospirillum brasilense* for the second reaction to address these issues. The residues in the binding sites for the substrates, 3-HPA and NAD^+^, were randomized, and the resulting libraries were screened for higher activity. Isolated KGSADH variants had significantly lower K_m_ values for both the substrates. The enzymes also showed higher substrate specificities for aldehyde and NAD^+^, less inhibition by NADH, and greater resistance to inactivation by 3-HPA than the wild-type enzyme. A recombinant *Pseudomonas denitrificans* strain with one of the engineered KGSADH variants exhibited less accumulation of 3-HPA, decreased levels of inactivation of the enzymes, and higher cell growth than that with the wild-type KGSADH. The flask culture of the *P. denitrificans* strain with the mutant KGSADH resulted in about 40% increase of 3-HP titer (53 mM) compared with that using the wild-type enzyme (37 mM).

## Introduction

**Figure 1:**
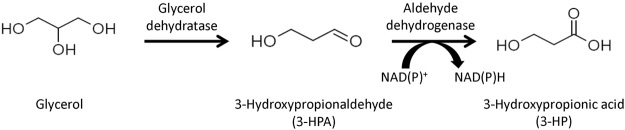
Biological conversion of glycerol to 3-HP via two successive enzymatic reactions.

3-Hydroxypropionic acid (3-HP) is an attractive renewable building block that can be produced from biomass. The presence of two functional groups, hydroxyl and carboxyl groups, allows its conversion into many useful chemicals^1,2^. For example, acrylic acid can be derived from 3-HP by catalytic reactions^3,4^. The global market for acrylic acid is expected to reach USD 22.55 billion by 2022 according to Grand View Research^5^. Biosynthetic pathways utilizing sugar^6–9^ or glycerol^10–16^ have been proposed and employed to develop metabolically engineered microorganisms to produce 3-HP. In particular, a metabolic pathway starting from glycerol (Fig. 1) has attracted much attention owing to the economic advantages stemming from the low price of crude glycerol (byproduct of biodiesel industry)^17–19^. Glycerol is converted into 3-hydroxypropanal (3-HPA) via a dehydration reaction catalyzed by glycerol dehydratase (GDHt), and the aldehyde intermediate 3-HPA is oxidized to 3-HP, along with the reduction of NAD(P)^+^ to NAD(P)H by aldehyde dehydrogenase (ALDH). Coenzyme B_12_-depedent glycerol dehydratase (DhaB) has been most widely used for the first reaction, despite the requirement of the expensive coenzyme B_12_^1,20,21^. To reduce or completely eliminate the need for coenzyme B_12_, several microorganisms that can naturally produce the vitamin have been tested for developing 3-HP producing recombinant strains including *Klebsiella pneumoniae* and *Pseudomonas denitrificans* ^22–25^. ALDH enzymes from diverse sources have been assessed for the conversion of 3-HPA to 3-HP^10,23,26–29^. However, none of them were designed in nature for the production of 3-HPA, and their relatively low activities have hampered the development of 3-HP-producing recombinant strains. Comparative studies for ALDHs were previously conducted, and α-ketoglutarate semialdehyde dehydrogenase (KGSADH) from *Azospirillum brasilense* was found a suitable ALDH enzyme for 3-HP production^27,30^.

There are several challenges associated with ALDH in developing recombinant microorganisms for an economically viable 3-HP production process. Among them, the toxicity of 3-HPA, cell death resulting from its accumulation, and the consequent cessation of 3-HP production have been well documented^31–34^. To prevent the accumulation of 3-HPA, the activity of ALDH (the second-step enzyme) was maintained at a higher level than that of GDHt (the first step enzyme), by balancing their expression levels^27,30^. A recent report described the optimization of the ratio between the two enzymatic reaction rates via UTR engineering^21^. A fed-batch cultivation of an engineered strain with the optimized pathway exhibited the higher 3-HP titer (40.51 g/L) and productivity (1.35 g/L/H) than that with the parental strain^14^. However, the strategy of controlling expression has a couple of limitations: the high expression of ALDH can be a burden on the host strain; the 3-HPA concentration cannot be decreased below a certain level which is dependent on the K_m_ of ALDH for 3-HPA. A more effective solution for addressing the 3-HPA toxicity would be engineering ALDH for higher activity, especially, by decreasing K_m_. Chu *et al*. reported an engineered ALDH, which exhibited 1.4-fold higher activity (78.07 U/mg) than the wild-type enzyme (55.12 U/mg), and a recombinant *E. coli* strain with the engineered enzyme showed about 20% increase of the 3-HP titer (71.9 g/L) in a 5 L bioreactor compared with the parental strain^29^. Another issue in glycerol-based 3-HP production is the regeneration of NAD(P)^+^. The redox balance in the cell affects various aspects of cellular physiology. The ratio of NAD(P)^+^ to NAD(P)H is adjusted and controlled based on the metabolic and environmental conditions. However, the extraneously introduced metabolic pathway converting NAD(P)^+^ to NAD(P)H could decrease the NAD(P)^+^/NAD(P)H ratio, especially in the late stages of 3-HP production, because of the toxic effects of 3-HPA and 3-HP on cellular physiology^1^. Anaerobic 3-HP production in the presence of nitrate was attempted to deal with the NAD(P)^+^ regeneration issue, and NAD(P)H accumulation can be alleviated by introducing NAD(P)H oxidation pathways^35^. However, engineering ALDH for lower K_m_ toward NAD(P)^+^ could be a more direct solution to address the low NAD(P)^+^ concentration. Based on these hypotheses, we attempted in this study, to engineer ALDH for improved activity toward 3-HPA and NAD(P)^+^ via lowering the K_m_ values for both the substrates. Specifically, KGSADH was chosen because the ALDH enzyme exhibited the highest 3-HP productivity among the ALDHs in the comparative analyses^30^. Directed evolutionary strategy^36–39^ was adopted as the engineering approach for modifying the substrate-binding sites of KGSADH. The engineered KGSADH enzymes were characterized with respect to various catalytic properties, including activities toward 3-HPA and NAD^+^, substrate specificities, product inhibition, 3-HPA inactivation, pH-dependence of activity, and temperature sensitivity. In addition, one of the variants was compared with the wild-type enzyme for 3-HP production from glycerol by a recombinant *Pseudomonas denitrificans* strain on flask scale.

## Results and Discussion

### Engineering the aldehyde-binding pocket of KGSADH

Initially, we targeted residues close to the active site of KGSADH. Since its structure was not available when we started the engineering, a homology model of the enzyme was constructed using the structure of human mitochondrial aldehyde dehydrogenase complexed with its aldehyde substrate, crotonaldehyde (PDB: 1O01)^40^, as template (Figures S1 and S2). The complex structure is one of several aldehyde dehydrogenase structures having their aldehyde substrates in the active site^40–42^, which helps to predict residues interacting with the aldehyde molecule in the modeled KGSADH structure. The comparison of its sequence with those of other aldehyde dehydrogenase enzymes had predicted E253 and C287 as the catalytic residues^43^. The superimposition of the template and the modeled KGSADH structures supported this prediction (Figure S1B). Based on the modeled structure, 10 positions close to the catalytic residues (Figure S3) were selected and randomized individually, using the NNK (K: G or T) degenerate codon, producing 10 libraries. Each library was screened (approximately 200 colonies) using the criterion of rate of formation of NADH from NAD^+^, by monitoring the increase in A_340_. Substrate concentrations chosen for 3-HPA and NAD^+^ were 1.5 mM and 1.0 mM respectively. The solution pH chosen for screening the libraries was 6. The rationale was to find variants tolerant to and/or showing an improved activity at low pH, because 3-HP production can lead to a decrease in cytoplasmic pH^1,44^. However, clones exhibiting higher activity than or comparable activity to the wild-type clone in cell lysate did not have any changes in the amino acid sequence (data not shown). The resistance to even small changes in the side chains suggests the critical roles of the 10 residues in catalysis and/or structure of KGSADH.

**Figure 2:**
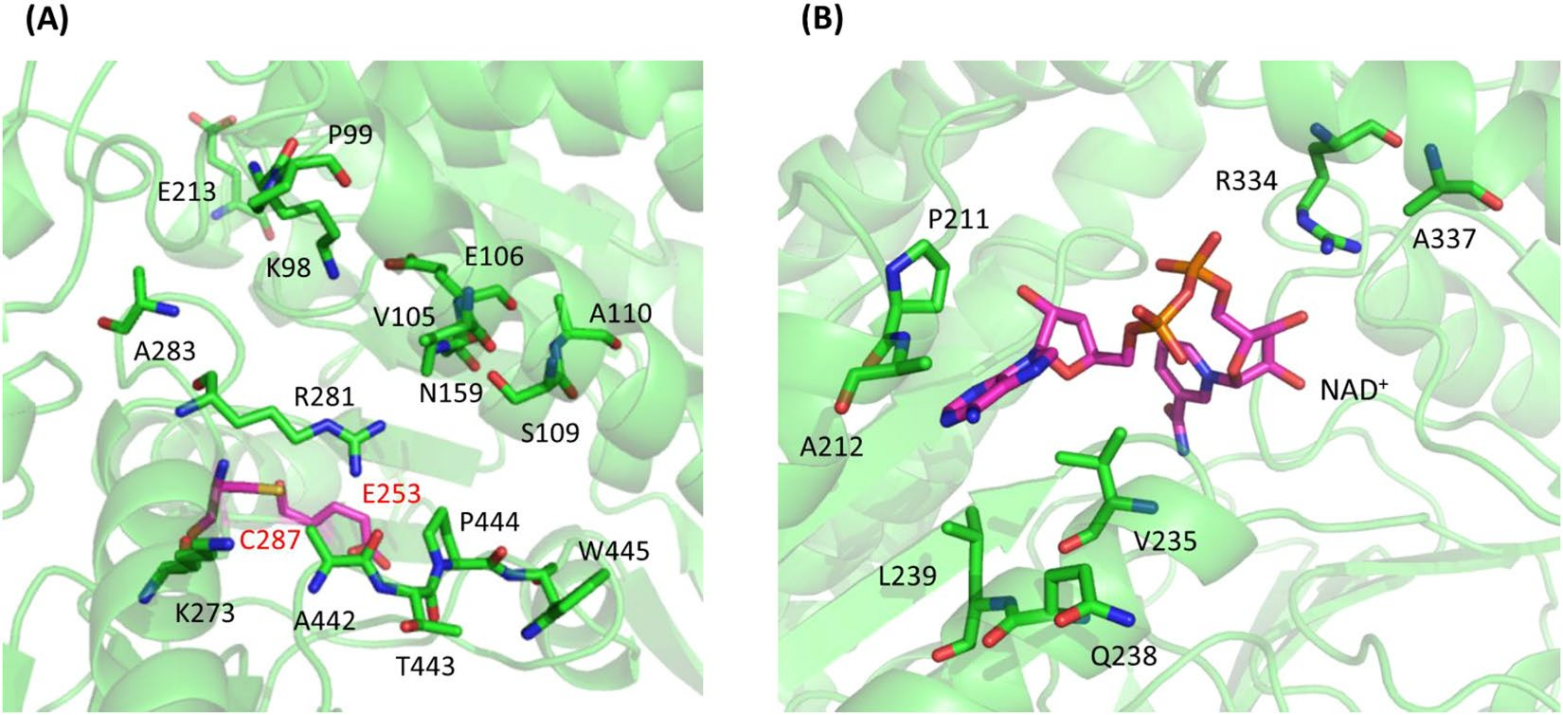
Target residues for randomization to generate libraries. (**A**) Residues for generating the single-site variant libraries of the aldehyde-binding site are shown using the KGSADH structure derived from homology modeling. Thirteen residues at the edge of the aldehyde-binding pocket and two residues (E215 and K273) based on the results of GabD4^29^ were chosen for randomization. The catalytic residues (C287 and E253) are shown in magenta. (**B**) Residues chosen for generating the NAD^+^ binding pocket library were shown using the crystal structure of KGSADH complexed with NAD^+^ (PDB:5X5U).

We then turned our focus to the residues in the aldehyde-binding pocket but having some distance to the catalytic residues. Based on the comparison of the template structure and the homology model of KGSADH, we selected 13 residues (Fig. 2A). Two more residues (E213 and K273) were chosen based on a report on the engineering of an aldehyde dehydrogenase from *Cupriavidus necator* (GabD4)^29^. In this study, two residues (E209 and E269) of GabD4 had been randomized for engineering the enzyme. We selected E213 and K273 of KGSADH as target positions, based on the sequence alignment of KGSADH with that of GabD4 (Figure S4). A total of 15 libraries were generated, in which the target residues were randomized using the NNK degenerate codon. Approximately 200 colonies were screened for each library. Eleven clones, the cell lysates of which exhibited similar or higher enzyme activity compared to that of the wild-type clone and included changes in the amino acid sequence, were purified and their kinetic properties of k_cat_ and K_m_ were characterized (Table S1). Several clones showed slightly higher activities (k_cat_/K_m_) toward either 3-HPA or NAD^+^ than the wild-type enzyme, but there was no variant having an enhanced activity for both the substrates. Interestingly, the N159V mutation significantly increased the K_m_ value toward 3-HPA, resulting in a 5.7-fold reduction of k_cat_/K_m_, but showed an enhanced activity toward NAD^+^, largely due to the decrease in K_m_. The crystal structure of KGSADH complexed with NAD^+^ was reported, while this manuscript was under preparation^45^. N159 is located close to the NAD^+^ binding pocket, but does not directly interact with the substrate (Figure S5). Actually, in the KGSADH structure paper, this residue was proposed to interact with 3-HPA. We suspect that the N159V mutation causes a relatively large structural perturbation in the substrate binding pockets.

An additional library was generated to investigate the combination of the mutations found in the aldehyde-binding site, and degenerate codons were used to replace a target position with multiple amino acids (Figure S6). The theoretical size of the library was 27648, and approximately 8000 colonies were examined using the same screening method. Three variants (designated 104, 106, and 108), whose cell lysates exhibited high activity, were isolated, and their amino acid sequences were determined (Table 1). These enzymes were purified, and their catalytic properties were characterized with respect to 3-HPA and NAD^+^ (Table 2). One variant (108) exhibited enhanced catalytic activity as demonstrated by the k_cat_/K_m_ value for 3-HPA. The K_m_ of variant 108 decreased more than 2-fold, compared to that of the wild-type enzyme. However, two enzymes (104 and 106) had lower k_cat_/K_m_ values than the wild-type enzyme toward 3-HPA. Interestingly, all three variants exhibited enhanced catalytic activities toward NAD^+^, in contrast to that toward 3-HPA, which was largely because of the increased k_cat_ values.

**Table 1:** Amino acid sequences of KGSADH enzymes.

Clone	Position*								
	110	159	273	281	334	337	442	443	444
KGSADH	A	N	K	R	R	A	A	T	P
104	**S**	N	**A**	R	R	A	**P**	T	**T**
106	A	N	K	R	R	A	**P**	T	**T**
108	A	N	**A**	R	R	A	**P**	E	**A**
WT-QR	A	N	K	R	**Q**	**R**	A	T	**P**
104-QR	**S**	N	**A**	R	**Q**	**R**	**P**	T	**T**
106-QR	A	N	K	R	**Q**	**R**	**P**	T	**T**
108-QR	A	N	**A**	R	**Q**	**R**	**P**	E	**A**

*The amino acid residues different from those in the wild-type KGSADH are shown in bold.

**Table 2:** Kinetic characterization of KGSADH enzymes at pH 8*.

Clone	3-HPA**			NAD^+^***			NADP^+^***		
	k_cat_ (s^−1^)	K_m_ (mM)	k_cat_/K_m_ (s^−1^ mM^−1^)	k_cat_ (s^−1^)	K_m_ (mM)	k_cat_/K_m_ (s^−1^ mM^−1^)	k_cat_ (s^−1^)	K_m_ (mM)	k_cat_/K_m_ (s^−1^ mM^−1^)
KGSADH	15 (±2.1)	1.6(±0.14)	9(±2.1)	12(±1.7)	0.21(±0.018)	57(±13.0)	8.6(±0.84)	2.3(±0.033)	3.7(±0.42)
104	15(±3.3)	2.2(±0.23)	7(±2.2)	19(±2.0)	0.28(±0.045)	68(±18.0)	—	—	—
106	12(±2.5)	1.4(±0.55)	9(±5.2)	20(±2.0)	0.27(±0.050)	74(±21.1)	—	—	—
108	11(±3.0)	0.78(±0.087)	14(±5.4)	18(±1.0)	0.27(±0.0045)	67(±4.81)	—	—	—
WT-QR	6.8(±0.35)	0.55(±0.13)	12(±3.6)	7.9(±0.75)	0.044(±0.0033)	180(±30.5)	4.4(±1.4)	0.81(±0.15)	5.4(±2.7)
104-QR	6.7(±0.64)	0.43(±0.094)	16(±4.9)	5.9(±0.61)	0.033(±0.0089)	179(±66.7)	4.7(±0.79)	0.70(±0.075)	6.8(±1.8)
106-QR	5(±1.2)	0.29(±0.062)	17(±7.8)	4.8(±0.93)	0.025(±0.0045)	192(±72.5)	4.0(±1.7)	0.54(±0.038)	7.2(±3.6)
108-QR	6(±1.3)	0.17(±0.032)	35(±14)	6.1(±0.64)	0.037(±0.0024)	165(±28.0)	4.5(±1.1)	0.66(±0.065)	6.9(±2.3)

*The experiments were repeated at least three times. **k_cat_ and K_m_ for 3-HPA were determined using 2 mM NAD^+^. ***k_cat_ and K_m_ for NAD^+^ and NADP^+^ were determined using 3 mM 3-HPA.

### Engineering the NAD^+^-binding pocket of KGSADH

Next, we targeted the NAD^+^ binding site of KGSADH, and its K_m_ for NAD^+^ was determined as 0.21 mM (Table 2). While the aldehyde-binding sites of ALDHs are as diverse as their substrates, the binding site for NAD^+^ of ALDHs has been known to be relatively conserved^46^. Therefore, we examined the interaction with NAD^+^, using the crystal structures of two ALDHs complexed with NAD^+^, whose K_m_ values were 0.047 mM and 0.0019 mM respectively (PDB: 1A4Z, 1BXS)^47,48^. Nineteen positions were identified, whose side chains are involved in the interactions with NAD^+^, and the residues of KGSADH for the positions were identified based on the sequence alignment (Table S2). Then, the amino acid residues at these positions of KGSADH were compared with those of ALDHs showing low K_m_ values toward NAD^+^^49–59^ (Table S2). Seven positions (P211, A212, V235, Q238, L239, R334, and A337) (Fig. 2B), which show variations as compared with the sequences of other ALDHs showing low K_m_ values, were selected and randomized, as shown in Figure S7. Even though some residues (P153 and Q160) in the NAD^+^-binding pocket of KGSADH are different from other ALDHs, they were tested earlier, when the residues close to the catalytic residues were targeted (Figure S3), and therefore were not included for making the NAD^+^ binding pocket libraries.

Two libraries were made using clones 104 and 106 as templates and were screened at low substrate concentrations (0.5 mM of 3-HPA and 0.3 mM of NAD^+^), with the objective of finding variants with reduced K_m_ values. Clones from both the libraries, which showed high activity in cell lysates, were isolated, and their amino acid sequences are shown in Figure S8. Clone 104-M5, exhibiting the highest activity, had two changes at positions R334 and A337; the two residues were mutated into Q and R respectively. Interestingly, two clones (clones 106-M4 and 106-M5) isolated from the 106 library also had the same changes (R334Q and A337R) in the targeted NAD^+^-binding site and another clone (clone 106-M8) showing a comparable activity to that of these two clones had one additional mutation at position 238 (Q238K) (Figure S8B). Based on these results, we decided to introduce the two mutations, R334Q and A337R, into the 108 variant also. The three enzymes (104-QR, 106-QR, and 108-QR; the sequences are shown in Table 1) were purified and their catalytic properties, k_cat_ and K_m_, toward 3-HPA and NAD^+^ were characterized (Table 2). The two mutations in the NAD^+^ binding pocket resulted in 7–11-fold reduction of K_m_ toward NAD^+^ for the three variants, and unexpectedly the K_m_ values for 3-HPA also decreased to a considerable extent. However, the k_cat_ also decreased. Therefore, the improvement in k_cat_/K_m_ was in the range of 2–4-fold. It is quite interesting that the mutations in the NAD^+^-binding pocket made such a dramatic effect on the binding of 3-HPA to the enzyme. The two mutations are located far from the 3-HPA-binding site. To examine the effect of the mutations R334Q and A337R in the absence of other mutations, these mutations were introduced into the wild-type enzyme, and the catalytic properties were characterized (Table 2). Similar to the three variant enzymes, the wild-type enzyme harboring R334Q and A337R mutations, named WT-QR, showed a decrease in the K_m_ values for both 3-HPA and NAD^+^, which suggests that the two mutations affect not only the NAD^+^ binding pocket, but also the binding of 3-HPA. However, variants found in this study showed higher activities (k_cat_/K_m_) than WT-QR, which indicates the contribution of other mutations, besides the two mutations in the NAD^+^ binding site, to the improvement of catalytic efficiency.

### Substrate specificity and product inhibition of KGSADH enzymes

**Figure 3:**
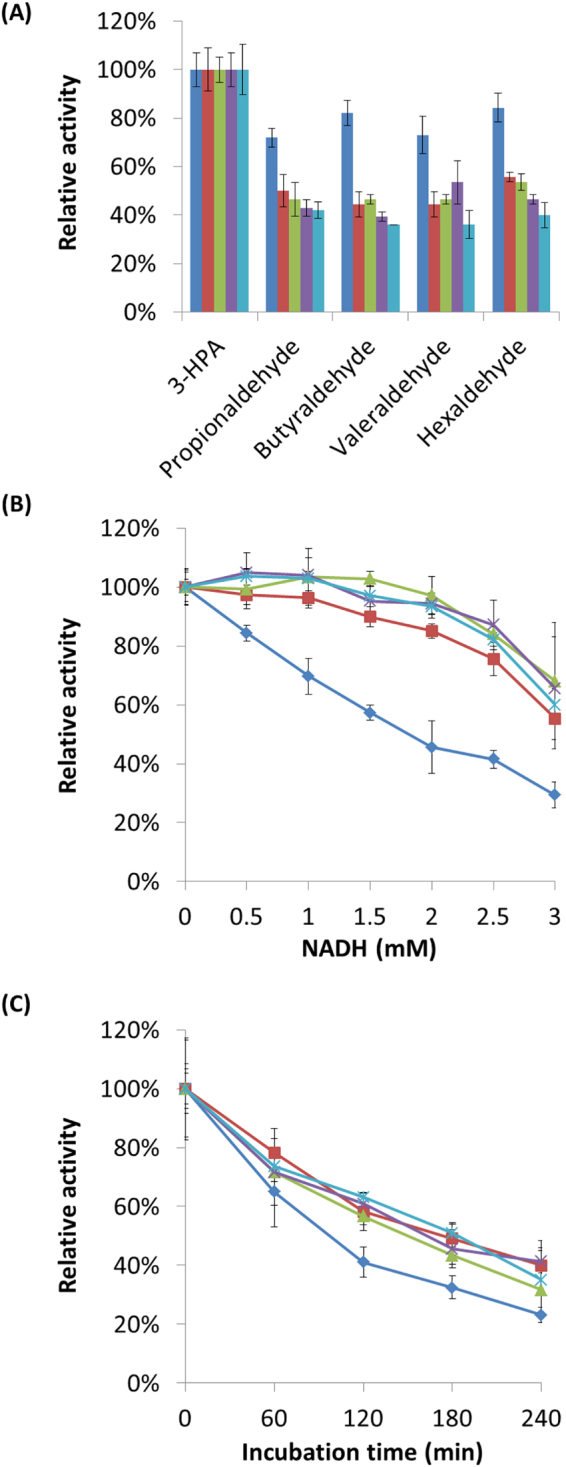
Characterization of the wild-type and the engineered KGSADH enzymes. (**A**) Activities of KGSADH enzymes toward several aldehydes, relative to that toward 3-HPA. (**B**) Product inhibition of KGSADH enzymes by NADH. The enzyme activity was measured in the presence of NADH at the indicated concentrations. (**C**) Inactivation of KGSADH enzymes by 3-HPA. Each of the variants was incubated with 20 mM 3-HPA for the indicated periods, and the residual activity at the end of the incubation was measured. The results for KGSADH are shown in blue, WT-QR in brown, 104-QR in green, 106-QR in violet, and 108-QR in cyan. All the experiments were repeated at least three times.

The wild-type KGSADH showed enzymatic activity toward various aldehyde substrates, in addition to its original substrate, α-ketoglutaric semialdehyde^30,60^. To examine how the mutations in the KGSADH variants affected the substrate specificity, their activities toward several aldehyde compounds were compared (Fig. 3A). All the variants showed lower relative activity toward other aldehydes, compared to that toward 3-HPA, than the wild-type enzyme, which indicates that the wild-type enzyme is more promiscuous toward aldehyde substrates than the engineered ones. In particular, the 108-QR variant exhibited the highest specificity, which might be attributed to its lowest K_m_ value toward 3-HPA, among all the tested enzymes. Interestingly, the WT-QR, which has mutations only in the NAD^+^ binding site, also exhibited increased specificity toward 3-HPA. The results, in addition to the decreased K_m_ for 3-HPA of WT-QR, suggest that the two mutations, R334Q and A337R, induce a structural change in the aldehyde-binding pocket, even though they are located away from the site. The KGSADH variants were characterized with respect to NADP^+^ also, because the wild-type enzyme was shown to be active with this cofactor (Table 2). The engineered enzymes had several-fold lower K_m_ values toward NADP^+^ and showed higher catalytic activity (k_cat_/K_m_) than the wild-type enzyme. However, the improvement toward NAD^+^ was higher than that for NADP^+^, and thus the activity of the engineered enzymes for NAD^+^ relative to those engineered for NADP^+^ was higher, compared to that of the wild-type enzyme.

Biological processes for the production of 3-HP, for which the engineered KGSADH variants are intended to be used, should generate high concentrations of 3-HP and NADH inside the recombinant microorganisms. Therefore, we investigated the reverse reaction of 3-HPA formation from 3-HP and product inhibitions by 3-HP and NADH. The highest concentration tested for 3-HP was 1 M, since the targeted titer of 3-HP production is approximately 100 g/L for developing an economically feasible process. The highest concentration chosen for NADH was 3 mM, considering the reported concentrations of NAD^+^ and NADH^61^. The reverse reaction did not take place up to 1 M 3-HP and 3 mM NADH, as determined by monitoring the absorbance at 340 nm (data not shown). However, product inhibitions were observed with both 3-HP and NADH. While there was no detectable inhibition by 3-HP up to 100 mM, the activities of KGSADH enzymes dropped approximately by 40% at 1 M 3-HP for all the tested KGSADH enzymes (Figure S9). Interestingly, the wild-type KGSADH exhibited much higher inhibition by NADH than the engineered enzymes (Fig. 3B). The inhibition was observed even at 0.5 mM NADH for the wild-type enzyme. The activity decreased linearly with increasing NADH concentration. In contrast, the variants showed less than 10% decrease in activity up to 2 mM NADH. The residual activity at 3 mM NADH was approximately 60%, compared to that in the absence of NADH. The concentration of NADH is rarely over 2 mM in the cytoplasm of microorganisms^62^. Therefore, the engineered KGSADH variants are not likely to experience inhibition by NADH during 3-HP production by recombinant microorganisms.

### pH sensitivity, 3-HPA inactivation, and heat inactivation of KGSADH enzymes

The screening of the libraries were conducted under the slightly acidic conditions, with the objective of finding variants tolerant to and/or showing improved activity at low pH, and the catalytic properties of the engineered enzymes were also characterized at pH 6 (Table S3). In general, the k_cat_ values decreased at low pH, whereas the K_m_ values did not change much. Interestingly, for the two variants, WT-QR and 106-QR, the K_m_ values for 3-HPA were lower at pH 6 than those at pH 8. The ratio of k_cat_/K_m_ value at pH 6 vs. pH 8 of these two variants was higher than that of other enzymes (Table S3). The two mutations, R334Q and A337R, in the NAD^+^ binding pocket might play a role in lowering the K_m_ for 3-HPA at acidic pH. However, it is not clear why only WT-QR and 106-QR exhibited the decreased K_m_ at pH 6, even though 104-QR and 108-QR have the same mutations. It is of note that 106-QR has two mutations near the aldehyde-binding site, whereas 104-QR and 108-QR have four mutations. Therefore, the structural difference between these two enzymes and WT-QR might be greater compared to that between WT-QR and 106-QR. The relative activities of the enzymes were determined in the pH range of 4–10 (Figure S10). These relative activities reflect k_cat_ rather than k_cat_/K_m_, considering the concentrations of the substrates used in this experiment (3 mM 3-HPA and 2 mM NAD^+^). The KGSADH enzymes showed similar pH profiles, with the exception of at pH 10. The activities of all the variants increased up to pH 10; the wild-type KGSADH had the highest activity at pH 9. The variant enzymes were more resistant to alkaline conditions than the wild-type enzyme.

3-HPA is a highly reactive compound and reacts with side chains of various amino acids, including those of Cys, Lys, His, and Arg^63,64^. Chemical modifications of the side chains can result in the loss of enzyme activity. We characterized the inactivation kinetics of the KGSADH variants (Fig. 3C). When incubated with a high concentration of 3-HPA (20 mM), the engineered enzymes exhibited higher resistance to 3-HPA inactivation than the wild-type KGSADH: the half-life values (t_1/2_) for the KGSADH variants, calculated using the fitted exponential decay, were at least 50% higher than that for the wild-type enzyme (Table S4). One possible reason for the resistance to alkaline condition and 3-HPA inactivation is the improved stability of the enzymes. We, therefore, compared the thermal stability of the enzyme variants (Figure S11 and Table S5). Contrary to our expectation, wild-type enzyme showed the highest resistance to heat inactivation, which is largely related to the structural stability. These results suggest that the tolerance to basic pH and resistance to inactivation by 3-HPA of the engineered enzymes do not arise from the thermodynamic stability, but might be related to the remodeled substrate binding sites, considering their correlation to the enhanced activity, altered substrate specificity, and decreased product inhibition by NADH.

### Structural modeling of the WT-QR variant

The results above indicated that the mutations in the NAD^+^ binding site, R334Q and A337R, affect various catalytic properties of KGSADH; K_m_ for both NAD^+^ and 3-HPA, substrate specificity, product inhibition by NADH, pH sensitivity, and resistance to 3-HPA inactivation. In order to investigate the effect of the two mutations, the structure of WT-QR was modeled using Molecular Operating Environment (MOE), based on the crystal structure of KGSADH complexed with NAD^+^^45^ (Figures S12 & S13). The two mutations, R334Q and A337R, result in new interactions with NAD^+^. In the wild-type enzyme, the guanidine group in the side chain of R334 interacts with the ribose ring close to the nicotinamide group via hydrogen bonding, but A337 does not show any interaction with NAD^+^ (Figure S12B). However, Q334 and R337 of the WT-QR variant interact with the phosphate group close to nicotinamide, resulting in structural changes in the region where the mutations are located (residue 325–337) and also conformational changes of NAD^+^ binding pocket (Figure S12B). The new interactions could contribute to the reduced K_m_ of WT-QR toward NAD^+^. Unexpectedly, in addition to the NAD^+^ binding site, the substrate entry regions of aldehyde-binding pocket (residues 101–109, 265–290 and 436–442) of WT-QR exhibit changes in the backbone structure, compared to that of wild-type structure (Figure S12C&S13). The peptide backbone for residues 441–445 moves inward, and thus the entrance size of aldehyde-binding pocket decreases, compared to that of wild-type enzyme (Figure S12C), which might explain the decreased K_m_ and the increased specificity toward 3-HPA. It is of note that the three variants (104, 106, and 108), isolated by screening the libraries constructed by targeting the 3-HPA-binding site, have mutations in residues 442, 443, or 444 (Table 1). While the two proteins showed nearly identical peptide backbone structures in the region close to the catalytic residues, conformational differences in the side chains were observed (Figure S12D), which might be associated with the decrease in k_cat_ of the mutant enzyme. Even though the modeled structure of WT-QR provides interesting insights into the effects of the two mutations, the results need to be confirmed by experimental work. Currently, we are trying to determine the crystal structures of the engineered KGSADH variants.

### Production of 3-HP from glycerol by a recombinant *P. denitrificans* strain on a flask scale

**Figure 4:**
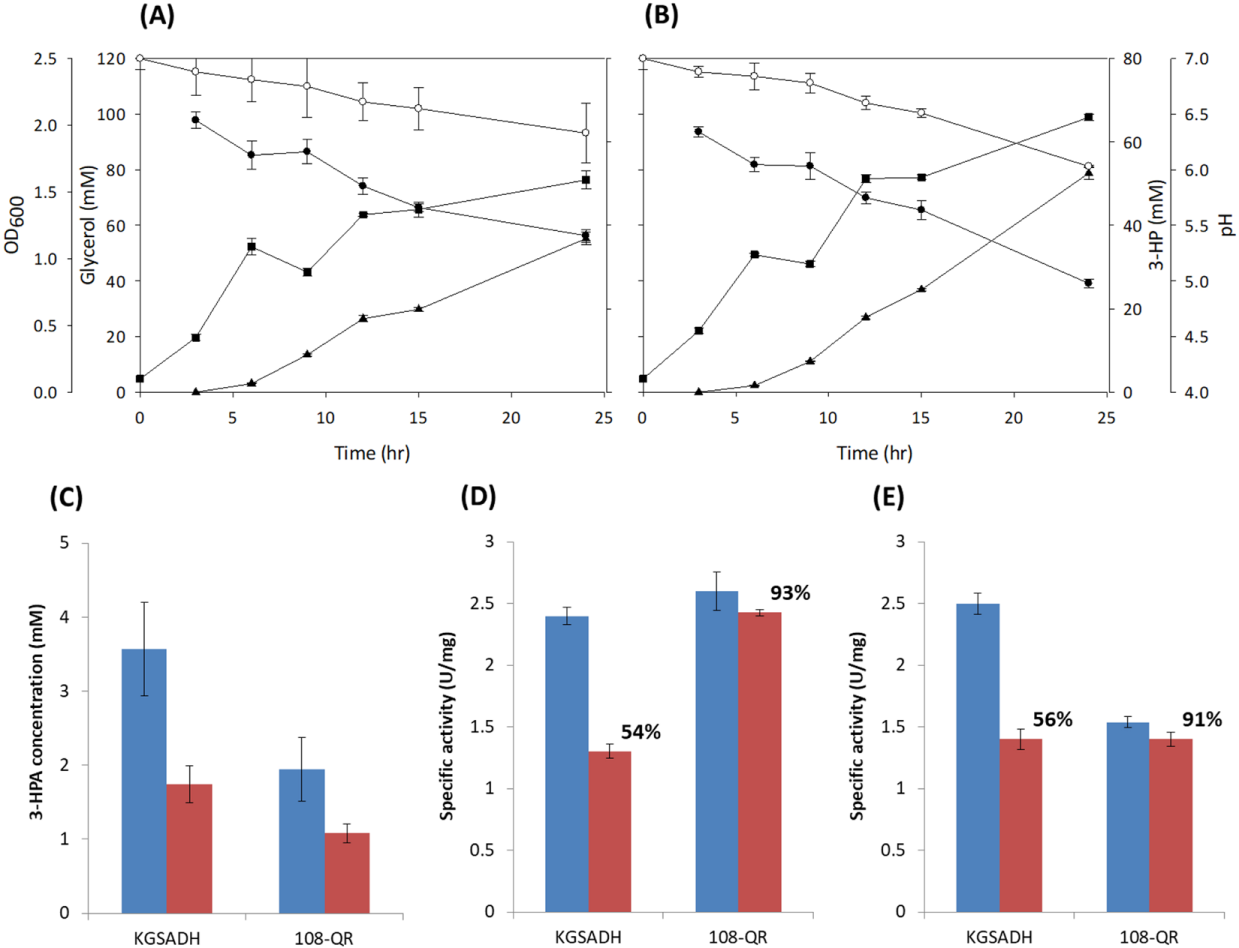
3-HP production from glycerol by recombinant *P. denitrificans* strains. (**A**,**B**) Time-course profiles of recombinant *P. denitrificans* Δ*3hpdH* Δ*3hibdhIV* with KGSADH (**A**) and recombinant *P. denitrificans* Δ*3hpdH* Δ*3hibdhIV* with 108-QR (**B**) cell growth (OD_600_, filled square), glycerol concentration (filled circle), 3-HP concentration (filled triangle), and pH (open circle) (**C**) Accumulation of 3-HPA in the culture media. 3-HPA concentration of two samples (12 h in blue and 24 h in brown) in the HPLC analyses were shown. (**D**) Activity of DhaB. (**E**) Activity of KGSADH. (**D**,**E**) Enzyme activities in the cell lysate were determined at two time points (12 h in blue and 24 h in brown). All the experiments were repeated twice.

We successfully engineered KGSADH for higher activity toward both 3-HPA and NAD^+^ via decreasing the K_m_ values. We were interested in investigating how the engineered enzymes performed in the production of 3-HP from glycerol by recombinant bacterial strains. The 108-QR enzyme showed the highest activity among the engineered enzymes, and we decided to compare the wild-type KGSADH with this variant. The *P. denitrificans* Δ*3hpdH* Δ*3hibdhIV* strain^65^ was transformed with the pUCPK plasmid^25^ for expressing glycerol dehydratase (DhaB), its reactivase (GdrAB), and KGSADH. The genes for DhaB and GdrAB are under the constitutive *bla* promoter^25^ and that for KGSADH, under the constitutive *3hibdhIV* promoter^63^. The culture experiments were conducted in duplicate for each enzyme, and the results were averaged. As shown in Fig. 4A and B, during the initial period of cultivation (3–12 hours), the 3-HP titer and the cell density were similar for the two recombinant *P. denitrificans* strains harboring wild-type KGSADH or 108-QR. However, in the late stage (15–24 hours), differences in 3-HP production and cell growth were observed. *P. denitrificans* with 108-QR exhibited a higher 3-HP titer (53 mM vs. 37 mM at 24 hours) and greater cell growth than *P. denitrificans* with the wild-type KGSADH (OD_600_ = 2.1 vs. OD_600_ = 1.6 at 24 hours) (Fig. 4A and B). The concentrations of 3-HPA were compared between the two strains at the two culture points (12 and 24 hours) (Fig. 4C). *P. denitrificans* with 108-QR showed ~50% lower 3-HPA accumulation at both the time points. The 3-HPA toxicity has been shown to be related to the inactivation of cellular enzymes, in particular, DhaB and KGSADH^33,66,67^. The reduced accumulation of this intermediate in *P. denitrificans* with 108-QR, results in less reduction of the activity of the two enzymes during the culture (Fig. 4D and E). In summary, the results described above indicate that the higher enzyme activities via decreased K_m_ enabled the higher cell growth, glycerol consumption, and 3-HP production. Further studies for the evaluation of the mutant KGSADH enzymes in culture experiments are under progress.

## Conclusion

In this study, we engineered the aldehyde dehydrogenase of KGSADH to address the issues in the biological production of 3-HP from glycerol using recombinant microorganisms: toxicity of the intermediate, 3-HPA, and low NAD^+^ regeneration efficiency in the late stages of culture. One of the approaches to tackle these problems could be improving the enzyme activity toward its substrates, 3-HPA and NAD^+^. KGSADH variants were isolated through screening various libraries targeting the substrate binding pockets. The engineered enzymes exhibited improved activity compared to the wild-type enzyme. In particular, the K_m_ of the enzymes for both the substrates decreased significantly. In addition, the KGSADH variants had several interesting properties: higher substrate specificities for aldehyde and the reducing cofactor; less product inhibition by NADH; and greater resistance to inactivation by 3-HPA. One of the engineered enzymes (108-QR) was compared with the wild-type KGSADH for 3-HP production in a recombinant *P. denitrificans* strain. The microorganism harboring the variant enzyme exhibited lower accumulation 3-HPA, less inactivation of the two enzymes that produce 3-HP from glycerol, higher cell growth, and higher 3-HP production than that with the wild-type enzyme. These phenomena were more pronounced in the late stage of culture. We expect that the problems of 3-HPA toxicity and NAD^+^ regeneration associated with developing economically feasible 3-HP production process could be addressed to some extent by using engineered aldehyde dehydrogenases. However, some of the properties of engineered enzymes can be further improved. These include the unintended decrease in k_cat_ and thermal stability observed in this work, product inhibition by 3-HP, 3-HPA inactivation, and activity under acidic conditions.

## Materials and Methods

### Materials

Pfu DNA polymerase was purchased from Solgent Co. Ltd. (Daejeon, KOREA). Restriction enzymes and T4 DNA ligase were purchased from New England BioLabs (Ipswich, MA, USA). Isopropyl β-D-1-thiogalactopyranoside (IPTG), Bovine Serum Albumin (BSA) and β-mercaptoethanol were purchased from BioShop (Burlington, ON, Canada). DNase, lysozyme, NAD^+^ and NADH were purchased from Sigma-Aldrich (St. Louis, Missouri, USA). B-PER reagent and Ni-NTA resin were purchased from Thermo Fisher Scientific (Waltham, MA, USA) and Clontech (Palo Alto, CA, USA) respectively. Centrifugal ultrafiltration filter (MWCO: 10,000) was purchased from Merck Millipore (Billerica, MA, USA). 3-HPA was synthesized chemically from acrolein, using the method described by Hall and Stern^68^. All other chemicals were of analytical grade and were used as received.

### Construction of libraries

Mutations were introduced into the KGSADH gene, using the assembly PCR method. The pQE80L/KGSADH plasmid^43^ was used as template. The primers used are shown in Table S6. The PCR products from primers 1 and 2 were ligated into the pQE80L plasmid, using the BamH1 and Pst1 sites. The resulting KGSADH libraries were transformed to *E. coli* DH10β. Primers 1–32 were used for constructing single-site variant libraries for the aldehyde-binding pocket, and primers 1, 2, and 33–42 for combining the beneficial mutations from the single-site variant libraries. Primers 1, 2, and 43–58 were used for the NAD^+^ binding site library.

### Screening of KGSADH libraries

Oxidation of 3-HPA to 3-HP is coupled with the reduction of NAD^+^ to NADH. Increase in absorbance at 340 nm (λ_max_ of NADH = 340 nm) was used to monitor the reaction catalyzed by KGSADH. Single colonies were picked from each library and inoculated into 96-well plates (U type). Cells were grown overnight in 100 µL 2xYT medium containing 200 µg/mL of ampicillin at 37 °C and then transferred into the same, but fresh media. When the OD_600_ approached 0.5, KGSADH expression was induced by adding 1 mM IPTG. After 6 h, the cells were harvested by centrifugation at 3,134 *g* and 4 °C for 45 min, and the cell pellets were stored at −80 °C until further use. The cell pellets were resuspended in 100 µL extraction buffer (100 mM phosphate buffer, pH 8, containing 10% B-PER, 100 µg/mL lysozyme, and 5 U/mL DNase), and incubated at 25 °C for 1 h. The cell lysates were centrifuged at 3,134 *g* and 4 °C for 45 min. Ten microliters of the supernatant was diluted with 170 µL activity assay buffer (100 mM phosphate buffer, pH 6, containing 1 mM EDTA, 1 mM DTT, and 1 mM β-mercaptoethanol), and the activity assay was initiated by adding the substrates, 3-HPA and NAD^+^. The rate of 3-HP formation was determined via the ΔA/min at 340 nm. To compensate the enzyme concentrations from different cell growth, the 3-HP production rate (ΔA/min) was divided by OD_600_ for analyzing the results.

### Expression and purification of KGSADH enzymes

The wild-type and mutant KGSADH enzymes in the pQE80L plasmid were expressed in *E. coli* DH10β cells. Cells were grown in 2xYT medium at 37 °C until OD_600_ reached 0.5, and then the expression of enzyme was induced by 1 mM IPTG. After 6 h, the cells were harvested by centrifugation at 9,300 *g* and 4 °C for 15 min, and the cell pellets were stored at −80 °C until further use. The KGSADH enzymes with the N-terminal His_6_-tag were purified using the Ni-NTA resin following manufacturer’s instructions. The protein buffer was exchanged with 10 mM phosphate buffer, pH 7.4, containing 150 mM NaCl, using the centrifugal ultrafiltration filter. The purified proteins were stored in the storage buffer (20 mM potassium phosphate, pH 7.5, containing 1 mM EDTA, 1 mM DTT, and 50% (v/v) glycerol). The concentrations of the purified proteins were determined by measuring the absorbance at 280 nm, using extinction coefficients calculated from the ProtParam site (http://web.expasy.org/protparam/).

### Characterization of KGSADH enzymes

k_cat_ and K_m_ values of the wild-type and variant enzymes were determined by measuring the reaction rate at 25 °C, by monitoring the increase in A_340_ (ε = 6,220 M^−1^ cm^−1^). To determine the kinetic constants for 3-HPA, the enzyme concentration was adjusted to 20 nM in 100 mM phosphate buffer (pH 8 or pH 6) containing 1 mM EDTA, 1 mM DTT, 0.2% BSA (w/v), and 2 mM NAD^+^. The reactions were initiated by the addition of varying concentrations of 3-HPA. For the k_cat_ and K_m_ values for NAD^+^ or NADP^+^, the enzymes were diluted with the buffer without NAD^+^ or NADP^+^, and the reactions were initiated by the addition of 3 mM 3-HPA and varying the concentrations of NAD^+^ or NADP^+^. The results were analyzed by nonlinear regression for the Michaelis-Menten equation, using Origin software (Northaempton, MA, USA). The relative activity of the enzyme toward various aldehyde substrates was determined by measuring the activity in 100 mM phosphate buffer, pH 8, containing 1 mM EDTA, 1 mM DTT, 0.2% BSA (w/v), 1.5 mM NAD^+^, and 1 mM aldehyde (3-HPA, propionaldehyde, butyraldehyde, valeraldehyde or hexaldehyde) at 25 °C^43^. The inhibition of KGSADH enzymes by 3-HP was determined by measuring the activity in 100 mM phosphate buffer, pH 8, containing 1 mM EDTA, 1 mM DTT, 0.2% BSA (w/v), 2 mM NAD^+^, and 1–1000 mM 3-HP. NADH inhibition was assayed in the same buffer containing 3 mM 3-HPA and 0–3 mM NADH. The effect of pH on the enzyme activity was investigated using actetate buffer (pH 4 and 5), phosphate buffer (pH 6–8), and glycine buffer (pH 9 and 10). The reactions were conducted in 100 mM buffer containing 1 mM EDTA, 1 mM DTT, 0.2% BSA (w/v), 2 mM NAD^+^, and 3 mM 3-HPA, at 25 °C. The inactivation of KGSADH by 3-HPA was investigated by measuring the residual activity after incubating the enzyme with 20 mM 3-HPA for varying lengths of time. The enzyme activity was determined in 100 mM phosphate buffer, pH 8, containing 1 mM EDTA, 1 mM DTT, 0.2% BSA (w/v), and 2 mM NAD^+^, at 25 °C. Heat inactivation of the enzymes was determined by measuring the residual activity after incubating the enzymes at 65 °C for varying lengths of time. The enzyme activity was determined in 100 mM phosphate buffer, pH 8, containing 1 mM EDTA, 1 mM DTT, 0.2% BSA (w/v), 2 mM NAD^+^, and 3 mM 3-HPA at 25 °C. The half-life (t_1/2_) values for inactivation by 3-HPA or heat treatment were determined by fitting the data to exponential decay (y = ae^bx^) using Origin software.

### Structural modeling of KGSADH enzymes

The three-dimensional structure of KGSADH was constructed by homology modeling, using the x-ray structure of human mitochondrial aldehyde dehydrogenase (PDB ID: 1O01) as the template^40^. The model was built using Molecular Operating Environment (MOE) and evaluated by PROCHECK and ProSA online structure analysis^69,70^. The protein structure was visually examined using PYMOL viewer (http://www.pymol.org).

The crystal structure of KGSADH complex with NAD^+^ (PDB ID: 5X5U) and apo form (PDB ID: 5X5 T) were retrieved form the Protein Data Bank and investigated for structural abnormalities and missing residues. The unbound solvent molecules and other nonstructural residues were removed to clean the structure for further structural analyses. The *In silico* structure of WT-QR complexed with NAD^+^ was generated via the residue scan module within MOE under the LowMode ensemble, in which the LowModeMD Search method generates mutant conformations using ~1 ps run of molecular dynamics simulation at constant temperature, followed by all-atom energy minimization under Amber10:EHT force field, as explained elsewhere^71^. Pymol and MOE were used for visual inspections and generating structural figures.

### Flask scale production of 3-HP

Recombinant *Psuedomonas denitrificans* was grown in an Erlenmeyer flask for comparing the performance of wild-type KGSADH with that of 108-QR. *P. denitrificans* Δ*3hpdH* Δ*3hibdhIV* ^65^, which does not degrade the produced 3-HP, was used as the host. The *dhaB- gdrAB* and *kgsadh* genes were cloned under the constitutive bla^25^ and 3hibdhIV^63^ promoters, respectively. Experiments were carried out in a working volume of 100 mL in a 250 mL non-baffled Erlenmeyer flask at 37 °C, on an orbital shaker incubator at 200 rpm. Modified M9 medium was used, which contained (per liter) 100 mmol potassium phosphate, 0.25 g MgSO_4_·7H_2_O, 1.0 g NaCl, 1.0 g NH_4_Cl, 1 g yeast extract, 2.0 g tryptone, 2.5 g glucose, 2.5 g L-glutamate, 30 mg kanamycin, 25 mg cobalt chloride, and 100 mmol glycerol. Inoculum culture was conducted under the same conditions as the main culture, except that glycerol was not added in the medium. Inoculum was cultured for 9 h to reach late exponential growth phase and added at ~0.01 OD_600_. The DhaB and KGSADH activities in the crude cell extract were measured using previously reported methods^20,72^. The 3-HPA concentrations were determined indirectly by measuring 1,3-propanediol using HPLC after enzymatic conversion of the aldehyde using alcohol dehydrogenase.

## Electronic supplementary material

Supplementary Information
