# Supplementary material for: Engineering an aldehyde dehydrogenase toward its substrates, 3-hydroxypropanal and NAD+, for enhancing the production of 3-hydroxypropionic acid
Source: Sci Rep. 2017 Dec 7;7:17155. doi: 10.1038/s41598-017-15400-x (PMC5719400; doi:10.1038/s41598-017-15400-x)
Supplement: Supplementary file 1 — Supplementary Information [file 41598_2017_15400_MOESM1_ESM.pdf]

Engineering an aldehyde dehydrogenase toward its substrates, 3-hydroxypropanal and NAD$^+$, for enhancing the production of 3-hydroxypropionic acid

Ye Seop Park,[1a] Un Jong Choi,[1a] Nguyen Hoai Nam,[2] Sang Jin Choi,[1] Abdul Nasir,[1] Sun-Gu Lee,[3] Kyung Jin Kim,[4] Gyoo Yeol Jung,[5] Sangdun Choi,[1] Jeung Yeop Shim,[6] Sunghoon Park,[2*] Tae Hyeon Yoo[1*]

[1] Department of Molecular Science and Technology, Ajou University, 206 World cup-ro, Yeongtong-gu, Suwon, 16499, Korea

[2] School of Energy and Chemical Engineering, Ulsan National Institute of Science and Technology (UNIST), Ulsan, 44919, Korea

[3] Department of Chemical and Biomolecular Engineering, Pusan National University, Pusan, 46241, Korea

[4] School of Life Sciences, Kyungpook National University, Daegu, 41566, Korea

[5] Department of Chemical Engineering, Pohang University of Science and Technology, Pohang, 37673, Korea

[6] Bio R&D Center, Noroo Holdings Co., Ltd., Suwon, 16299, Korea

[a]YSP and UJC contributed equally to this work.

* Correspondence should be addressed to

Sunghoon Park, Ph.D.

Tel: +82-52-217-2565

E-mail: parksh@unist.ac.kr

Tae Hyeon Yoo, Ph.D.

Tel: +82-31-219-3543

E-mail: taehyeonyoo@ajou.ac.kr

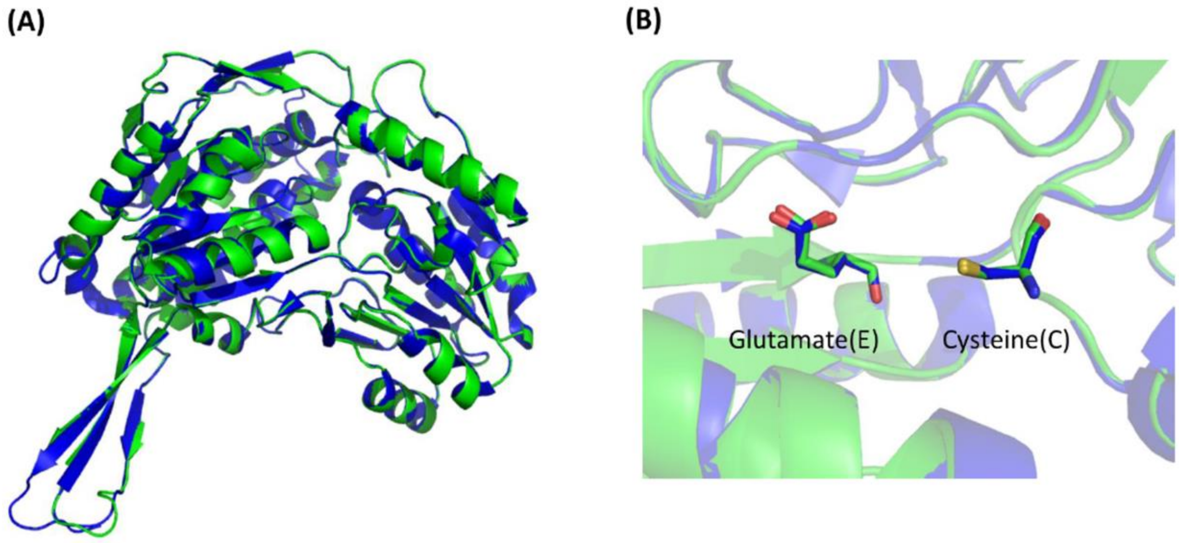

Figure S1. Homology modeled structure of KGSADH. (A) Superposition of the modeled KGSADH and human mitochondrial aldehyde dehydrogenase (PDB: 1O01) used as the template for homology modeling. (B) Zoomed-in view of the catalytic residues, Glu and Cys, in the active sites. The modeled KGSADH is shown in green, and the human mitochondrial aldehyde dehydrogenase in blue.

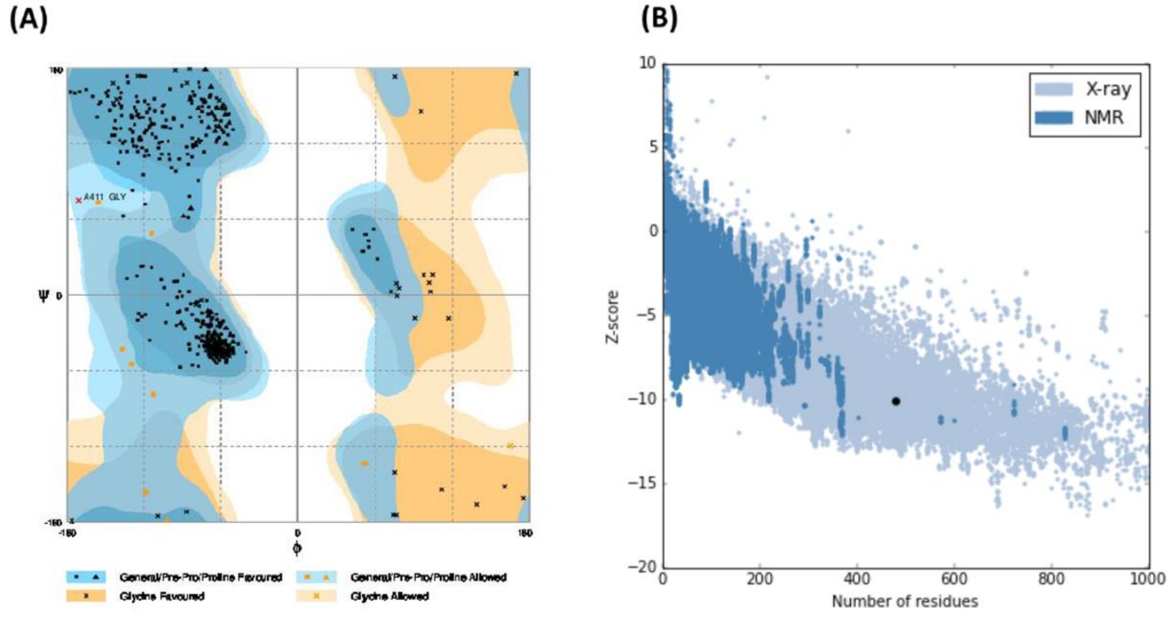

Figure S2. (A) Ramachandran plot of the Ψ/Φ distribution in KGSADH homology model. 90.4% residues were in the most favored regions, and 8.4% residues were in allowed regions. (B) Protein model quality score (Z-score) of KGSADH homology model is represented by the large black dot in the plot. Z-scores of all proteins in the Protein Data Bank as determined by X-ray crystallography (light blue-grey) and by nuclear magnetic resonance spectroscopy (dark blue-grey) are shown.

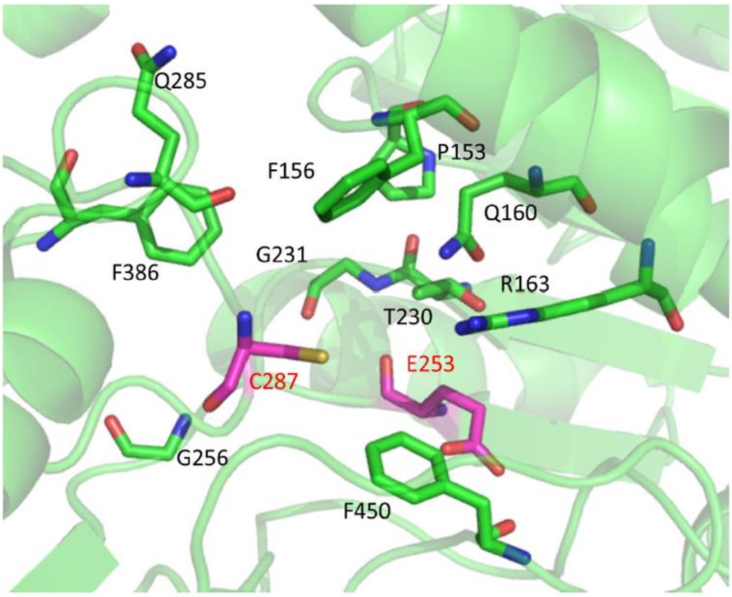

Figure S3. Residues close to the active sites (C287 and E253 in magenta) are shown using the homology modeled KGSADH structure.

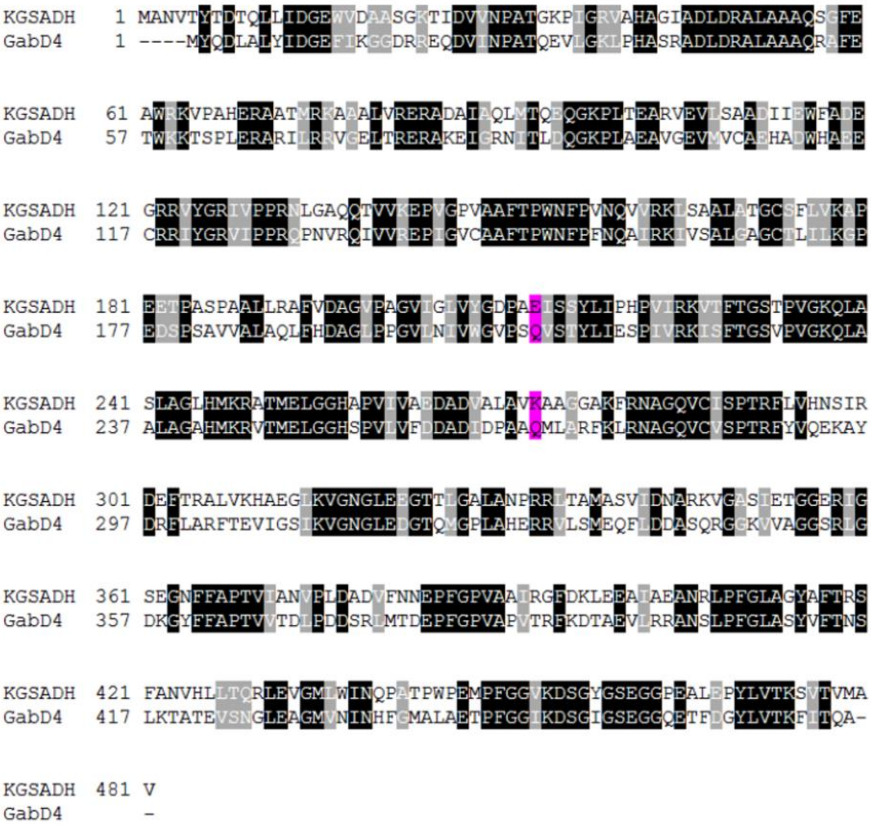

Figure S4. Amino acid sequence alignment of KGSADH and GabD4. The amino acid sequences of the two proteins were aligned using Box(http://www.ch.embnet.org/software/BOX_form.html). Identical and similar residues are shaded in black and gray, respectively. The two positions (E209 and E269) targeted in the GabD4 engineering[1] are shown in magenta (E213 and K273 in KGSADH).

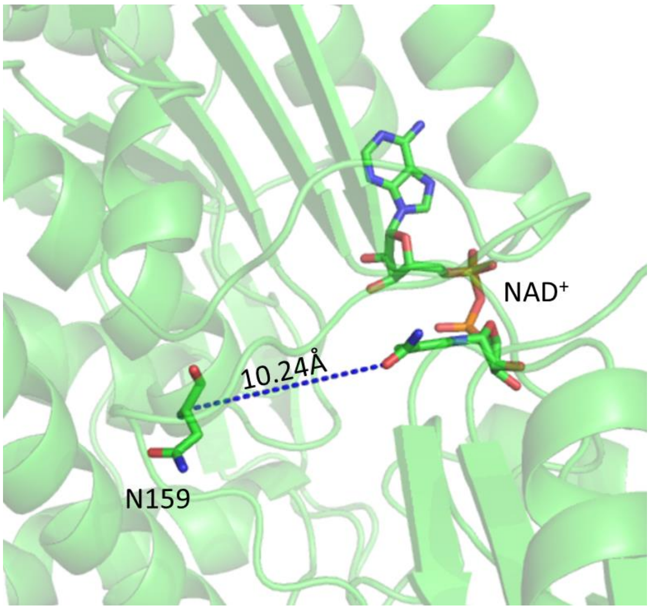

Figure S5. The relative positions of N159 and NAD^+ and the distance between the two are shown using the structure of KGSADH complexed with NAD^+ (PDB: 5X5U).

(A)

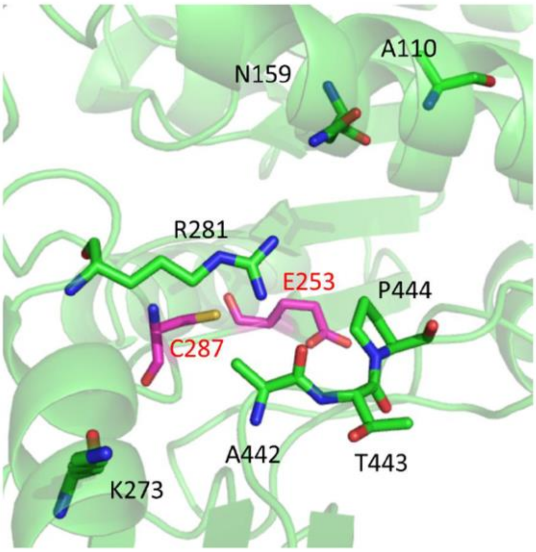

(B)

Position	Mutation (a.a)
A110	A/S
N159	N/A/V/I/T/D
K273	K/A/E/S/T/ochre
R281	R/Q/H
A442	A/P
T443	T/D/E/A/N/K
P444	P/E/T/Q/K/A

Figure S6. (A) Residues selected for generating a library to combine the mutations found by screening the single-site libraries of the aldehyde-binding site. (B) Amino acids introduced at each position. The unintended amino acids introduced because of using degenerate codons are shown in red.

Position	Mutation (a.a)
P211	P/G
A212	A/P
V235	V/I/T/A
Q238	Q/I/L/K
L239	L/I/V
R334	R/Q/H
A337	A/K/R

Figure S7. Amino acids introduced at each position for generating the NAD$^+$-binding site library. The unintended amino acid introduced because of using degenerate codons is shown in red.

(A)

	211	212	235	238	239	334	337	Relative activity
104	P	A	V	Q	L	R	A	
104-M1	P	A	V	Q	I	R	K	100%
104-M2	P	A	T	K	I	Q	R	107%
104-M3	A	P	I	K	I	Q	R	66%
104-M4	P	A	I	K	I	Q	K	128%
104-M5	P	A	V	Q	L	Q	R	153%
104-M6	P	A	T	I	I	Q	K	118%
104-M7	P	A	I	K	I	Q	K	127%
104-M8	P	A	I	Q	I	Q	K	116%

(B)

	211	212	235	238	239	334	337	Relative activity
106	P	A	V	Q	L	R	A	
106-M1	P	A	V	L	I	Q	K	156%
106-M2	P	A	V	K	I	R	A	169%
106-M3	P	A	T	K	I	Q	K	144%
106-M4	P	A	V	Q	L	Q	R	216%
106-M5	P	A	V	Q	L	Q	R	206%
106-M6	P	A	I	K	I	R	A	148%
106-M7	P	A	V	Q	L	R	A	128%
106-M8	P	A	V	K	L	Q	R	233%
106-M9	P	A	V	Q	L	R	A	116%

Figure S8. Screening results for the NAD^+ binding pocket libraries. Amino acid sequences and relative activities of selected clones are shown for the two libraries generated using clones 104 (A) and 106 (B) as templates. Amino acid sequences different from those in the templates are shown in gray cells.

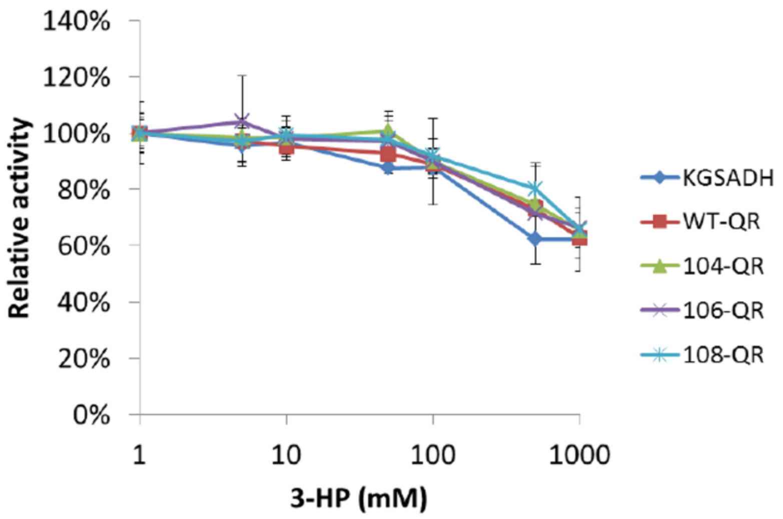

Figure S9. Product inhibition of KGSADH enzymes by 3-HP. The activities of the enzymes were measured in the presence of 3-HP at the indicated concentrations. The experiments were repeated at least three times.

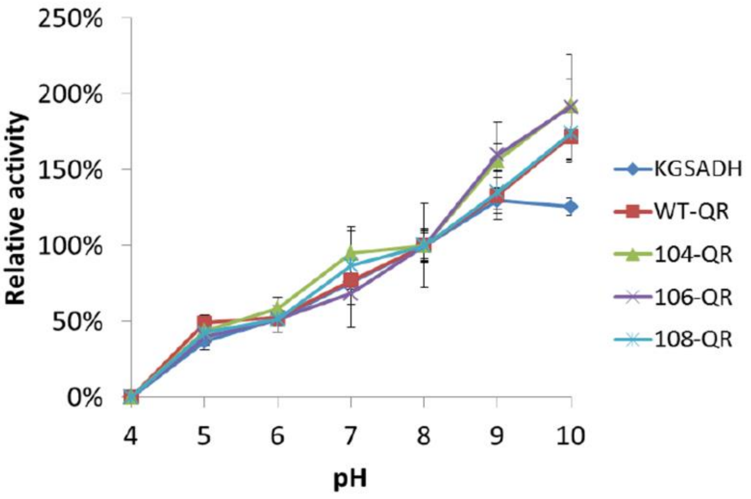

Figure S10. Activities of KGSADH enzymes at various pH. The activities of the enzymes were measured using acetate buffer (pH 4 and 5), phosphate buffer (pH 6-8), and glycine buffer (pH 9 and 10). The activities shown are relative to that of each enzyme at pH 8. The experiments were repeated at least three times.

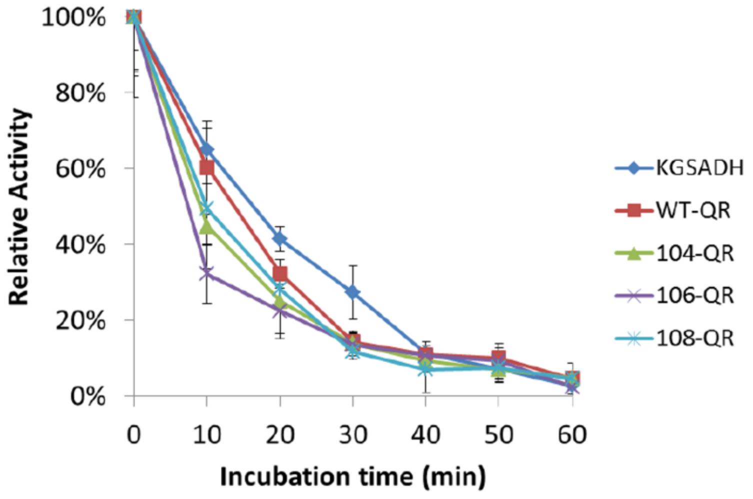

Figure S11. Inactivation of KGSADH enzymes by heat treatment. Each enzyme was incubated at 65 °C for the indicated periods, and the residual activities were measured after incubation. The experiments were repeated at least three times.

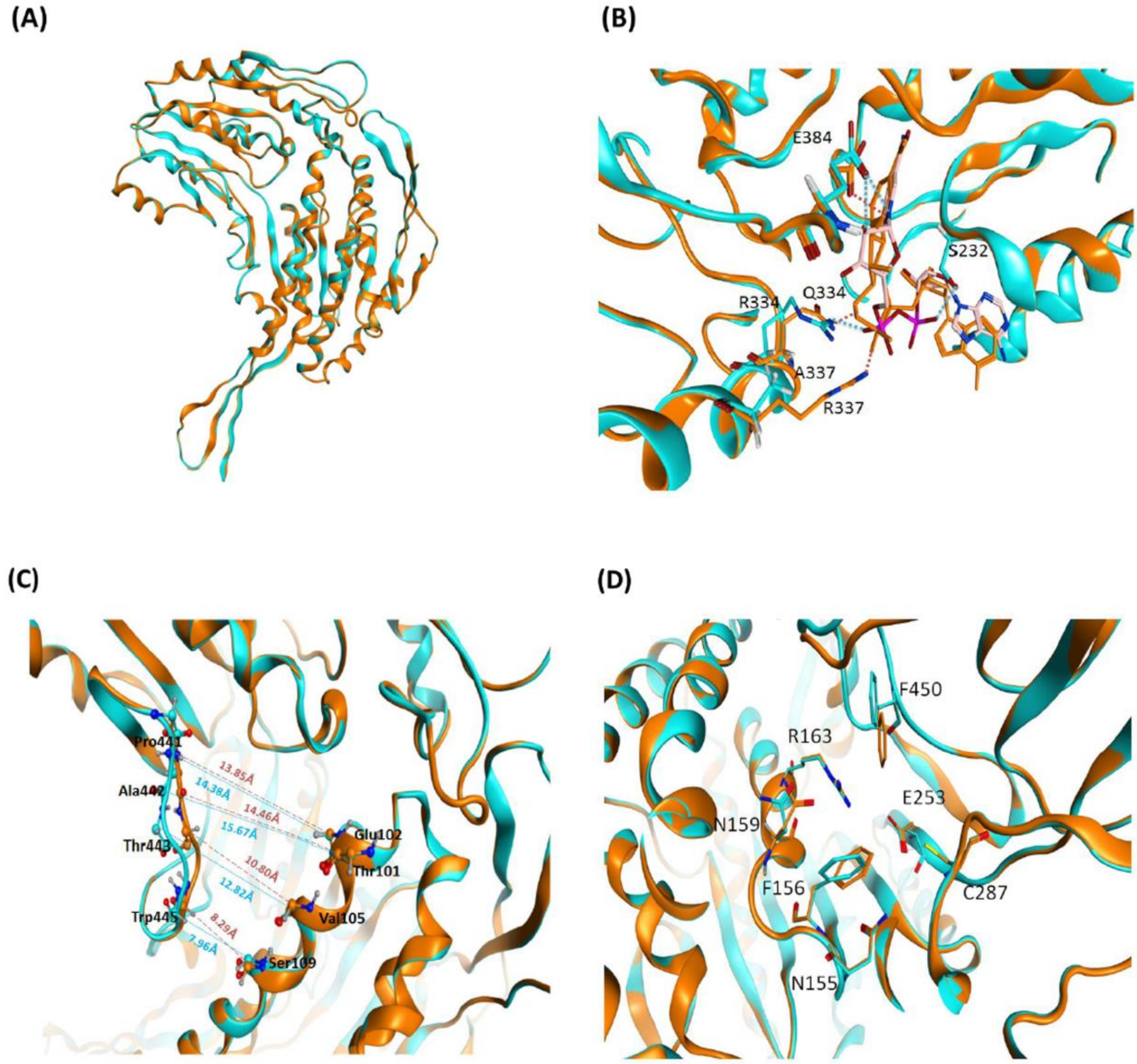

Figure S12. Modeled structure of the WT-QR variant. (A) Superposition of the KGSADH structure (PDB:5X5U) and the modeled structure of WT-QR. (B) Zoomed-in view for the interactions of NAD^+ in the binding pocket. (C) The entrance edge of the aldehyde-binding pocket. The distances between the loop and the α-helix located at the aldehyde-binding pocket entrance are shown. (D) Orientation of the residues close to the active sites. The KGSADH structure is shown in cyan, and the modeled structure of WT-QR in orange.

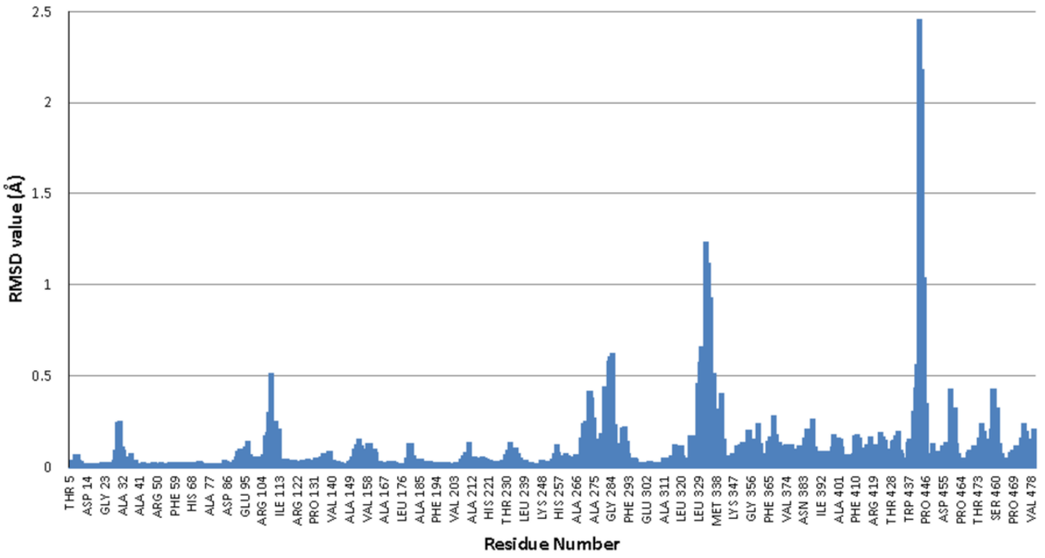

Figure S13. RMSD values for Cα atoms between the KGSADH structure (PDB: 5X5U) and the modeled WT-QR structure.

Table S1. Kinetic characterization of KGSADH enzymes*

Enzyme	3-HPA**			NAD^+***		
	k_{cat} (s^{-1})	K_m (mM)	k_{cat}/K_m (s^{-1} mM^{-1})	k_{cat} (s^{-1})	K_m (mM)	k_{cat}/K_m (s^{-1} mM^{-1})
KGSADH	15(±2.1)	1.6(±0.14)	9.4(±2.1)	12(±1.7)	0.21(±0.018)	57(±13.0)
A110S	16(±7.3)	1.8(±0.73)	8.9(±7.7)	10(±1.0)	0.20(±0.045)	50(±16.3)
N159V	11(±6.1)	6.8(±3.89)	1.6(±1.8)	6(±1.7)	0.07(±0.025)	86(±54.9)
K273A	16(±5.8)	2.0(±0.98)	8.0(±6.8)	12(±2.1)	0.16(±0.044)	75(±33.8)
K273E	14(±4.3)	1.6(±0.67)	8.8(±6.4)	13(±3.1)	0.24(±0.026)	54(±18.8)
K273S	16(±3.4)	1.7(±0.42)	9.4(±4.3)	11(±0.6)	0.16(±0.023)	69(±13.6)
R281Q	13(±3.3)	1.3(±0.55)	10(±6.8)	9(±0.2)	0.14(±0.010)	64(±6.0)
A442P	18(±3.6)	1.8(±0.60)	10(±5.3)	11(±2.2)	0.19(±0.0002)	58(±11.6)
T443D	15(±4.9)	1.2(±0.32)	13(±7.4)	12(±3.4)	0.22(±0.052)	55(±28.3)
T443E	22(±6.3)	2.1(±0.44)	11(±5.2)	16(±1.9)	0.28(±0.061)	57(±19.2)
P444E	11(±3.8)	1.1(±0.08)	10(±4.2)	10(±1.0)	0.17(±0.008)	59(±8.7)
P444T	18(±6.3)	1.7(±0.44)	11(±6.4)	13(±1.8)	0.23(±0.039)	57(±17.4)

*The experiments were repeated at least three times.

**k_{cat} and K_m for 3-HPA were determined using 1 mM NAD^+.

***k_{cat} and K_m for NAD^+ were determined using 1.5 mM 3-HPA.

Table S2. Amino acid sequence alignment of aldehyde dehydrogenases

Enzyme	Source	Km for NAD$^+$ (mM)	Position*																			Ref
			153	154	155	160	178	181	211	212	230	232	235	238	239	253	334	337	384	386	450	
KGSADH	*Azospirillum brasilense*	0.21	P	W	N	Q	K	E	P	A	T	S	V	Q	L	E	R	A	E	F	F	
ALDH	*Ovis aries* liver cytosol	0.0019	P	W	N	M	K	E	G	P	T	S	V	V	I	E	Q	K	E	F	F	2
ALDH	*Bos taurus* liver cytosol	0.047	P	W	N	M	K	E	G	P	T	S	V	L	I	E	Q	K	E	F	F	3
ALDH3	*Homo sapiens*	0.004	T	W	N	L	K	E	V	P	T	S	V	I	I	E	H	R	E	F	F	4
ALDH3A1	*Homo sapiens* saliva	0.0012	T	W	N	L	K	E	V	P	T	S	V	I	I	E	H	R	E	F	F	5
ALDH1B1	*Homo sapiens* mitochondria	0.0036	T	W	N	M	K	E	G	P	T	S	V	V	I	E	Q	R	E	F	F	6
ALDH2*2	*Homo sapiens* mitochondria	0.007	P	W	N	M	K	E	G	P	T	S	I	I	I	E	Q	K	E	F	F	7
ALDH1	*Homo sapiens* cytosol	0.0145	P	W	N	M	K	E	G	P	T	S	V	V	I	E	Q	K	E	F	F	8
ALDH2	*Homo sapiens* mitochondria	0.026	P	W	N	M	K	E	G	P	T	S	I	I	I	E	Q	K	E	F	F	9
ALDH10	*Spinacia oleracea*	0.032	P	W	N	M	K	E	G	P	T	S	T	T	V	E	Q	K	E	F	W	10
Phenyl acetaldehyde dehydrogenase	*Escherichia coli*	0.035	P	W	N	L	K	E	G	H	T	S	T	T	L	E	H	S	E	F	F	11
ALDH	Rat liver cytosol	0.038	P	W	N	M	K	E	G	P	T	S	V	V	I	E	Q	K	E	F	F	12
ALDH	*Pseudomonas aeruginosa*	0.044	P	W	N	S	K	E	G	A	T	G	T	T	I	E	H	S	E	F	F	13
ALDH2 RF2C	*Zea mays*	0.044	P	W	N	M	K	E	G	P	T	S	V	V	V	E	Q	K	E	F	F	14

* The amino acid position is based on the sequence of KGSADH.

Table S3. Kinetic characterization of KGSADH enzymes at pH 6*

Enzyme	3-HPA**				NAD$^+$***			
	k_{cat} (s^{-1})	K_m (mM)	k_{cat}/K_m (s^{-1} mM^{-1})	$(k_{cat}/K_m)_{pH\ 6}$/ $(k_{cat}/K_m)_{pH\ 8}$	k_{cat} (s^{-1})	K_m (mM)	k_{cat}/K_m (s^{-1} mM^{-1})	$(k_{cat}/K_m)_{pH6}$/ $(k_{cat}/K_m)_{pH8}$
KGSADH	7.6(±1.23)	1.4(±0.24)	5.4(±1.8)	0.58	5.1(±1.36)	0.18(±0.032)	28(±12.6)	0.50
104	7.5(±1.77)	1.6(±0.10)	4.7(±1.4)	0.69	7.9(±0.79)	0.23(±0.034)	34(±8.51)	0.51
106	6.6(±1.46)	1.3(±0.51)	5.1(±3.1)	0.59	8.3(±0.38)	0.22(±0.021)	38(±5.33)	0.51
108	5.9(±0.82)	0.87(±0.13)	6.8(±2.0)	0.48	7.9(±0.43)	0.26(±0.063)	30(±9.02)	0.46
WT-QR	2.4(±0.40)	0.29(±0.15)	8.3(±5.7)	0.67	3.4(±0.25)	0.041(±0.002)	83(±10.1)	0.46
104-QR	3.7(±0.37)	0.40(±0.07)	9.3(±2.6)	0.59	3.1(±0.27)	0.031(±0.007)	100(±31)	0.56
106-QR	2.5(±0.67)	0.17(±0.07)	14(±9.6)	0.85	2.4(±0.32)	0.023(±0.005)	104(±37)	0.54
108-QR	3.0(±0.49)	0.14(±0.02)	21(±6.7)	0.61	2.9(±0.20)	0.028(±0.008)	104(±37)	0.63

*The experiments were repeated at least three times.

**k_{cat} and K_m for 3-HPA were determined using 2 mM NAD$^+$.

***k_{cat} and K_m for NAD$^+$ were determined using 3 mM 3-HPA.

Table S4. Half-life for inactivation of KGSADH enzymes by 3-HPA

	KGSADH	WT-QR	104-QR	106-QR	108-QR
$t_{1/2}$ (min)	105.34	173.72	146.54	174.60	172.42

Table S5. Half-life for inactivation of KGSADH enzymes by heat treatment

	KGSADH	WT-QR	104-QR	106-QR	108-QR
$t_{1/2}$ (min)	14.67	12.42	10.10	8.43	10.53

Table S6. Primers used in the study

No.	Primer	Sequence
1	KGSADH library F	GATTCAATTGTGAGCGGATAAC
2	KGSADH library R	CTTCCTTAGCTCCTGAAAATCTCG
3	KGSADH 213 F	GGCGATCCGGCCNNKATCTCGTCGTACCTG
4	KGSADH 213 R	CAGGTACGACGAGATNMMGGCCGGATCGCC
5	KGSADH 273 F	GTTGCGCTCGCGGTGNNKGCGGCCGGCGG
6	KGSADH 273 R	CCGCCGGCCGCMNNCACCGCGAGCGCAAC
7	KGSADH 106 F	CCGAAGCGCGCGTCNNKGTGCTGTCGGCGG
8	KGSADH 106 R	CCGCCGACAGCACMNNGACGCGCGCTTCGG
9	KGSADH 110 F	GTCGAAGTGCTGTCGNNKGCGGACATCATC
10	KGSADH 110 R	GATGATGTCCGCMNNCGACAGCACTTCGAC
11	KGSADH 159 F	GTGGAATTTCCCGGTCNNKCAGGTCGTGCGCAAG
12	KGSADH 159 R	CTTGCGCACGACCTGMNNGACCGGGAAATTCCAC
13	KGSADH 281 F	GGCGGCGCGAAGTTCNNKAACGCGGGGCAGG
14	KGSADH 281 R	CCTGCCCCGCGTTMNNGAACTTCGCGCCGCC
15	KGSADH 283 F	GAAGTTCCGCAACNNKGGGCAGGTCTGCATC
16	KGSADH 283 R	GATGCAGACCTGCCCMNNGTTGCGGAACTTC
17	KGSADH 442 F	GATCAACCAGCCGNNKACGCCGTGGCCGGAAATG
18	KGSADH 442 R	CATTTCCGGCCACGGCGTMNNCGGCTGGTTGATC
19	KGSADH 443 F	GATCAACCAGCCGGCGNNKCCGTGGCCGGAAATG
20	KGSADH 443 R	CATTTCCGGCCACGGMNNCGCCGGCTGGTTGATC
21	KGSADH 444 F	CAACCAGCCGGCGACGNNKTGGCCGGAAATGCC
22	KGSADH 444 R	GGCATTTCCGGCCAMNNCGTCGCCGGCTGGTTG
23	KGSADH 445 F	CAGCCGGCGACGCCGNNKCCGGAAATGCCGTTC
24	KGSADH 445 R	GAACGGCATTTCCGGMNNCGGCGTCGCCGGCTG
25	KGSADH 98 F	GAGCAGGGCNNKCCGCTCACCGAAGCG
26	KGSADH 98 R	CGCTTCGGTGAGCGGMNNGCCCTGCTC
27	KGSADH 99 F	CAGGAGCAGGGCAAGNNKCTCACCGAAG
28	KGSADH 99 R	CTTCGGTCAGMNNCTTGCCCTGCTCCTG
29	KGSADH 105 F	CACCGAAGCGCGCNNKGAAGTGCTGTC
30	KGSADH 105 R	GACAGCACTTCMNNGCGCGCTTCGGTG
31	KGSADH 109 F	GTCGAAGTGCTGNNKGCGGCGGACATC
32	KGSADH 109 R	GATGTCCGCCGCMNNCAGCACTTCGAC
33	KGSADH A110A/S F	CGAAGTGCTGTCGKCCGCGGACATCATCG
34	KGSADH A110A/S R	CGATGATGTCCGCGGMCGACAGCACTTCG
35	KGSADH N159N/A/V F	GTGGAATTTCCCGGTCRHCCAGGTCGTGCGCAAG
36	KGSADH N159N/A/V R	CTTGCGCACGACCTGGDYGACCGGGAAATTCCAC
37	KGSADH K273K/A/S/E F	GTTGCGCTCGCGGTGDMAGCGGCCGGCGG
38	KGSADH K273K/A/S/E R	CCGCCGGCCGCTKHCACCGCGAGCGCAAC
39	KGSADH R281R/Q F	GGCGGCGCGAAGTTCCRKAACGCGGGGCAGG
40	KGSADH R281R/Q R	CCTGCCCCGCGTTMYGGAACTTCGCGCCGCC
41	KGSADH A442T443P444 F	GCTGTGGATCAACCAGCCGSCGRMWVMATGGCCGGAAATGCCGTTCG
42	KGSADH A442T443P444 R	CGAACGGCATTTCCGGCCATKBWKYCGSCGGCTGGTTGATCCACAGC
43	P211A212 PA F	GATCGGCCTCGTGTACGGCGATCCGGCCGAAATCTCGTCGTACCTG

44	P211A212 PA R	CAGGTACGACGACATTTCGGCCGGATCGCCGTACACGAGGCCGATC
45	P211A212 PP F	GATCGGCCTCGTGTACGGCGATCCGCCGGAAATCTCGTCGTACCTG
46	P211A212 PP R	CAGGTACGACGAGATTTCCGGCGGATCGCCGTACACGAGGCCGATC
47	P211A212 GA F	GATCGGCCTCGTGTACGGCGATGGCGCCGAAATCTCGTCGTACCTG
48	P211A212 GA R	CAGGTACGACGAGATTTCGGCGCCATCGCCGTACACGAGGCCGATC
49	P211A212 GP F	GATCGGCCTCGTGTACGGCGATGGCCCGGAAATCTCGTCGTACCTG
50	P211A212 GP R	CAGGTACGACGAGATTTCCGGGCCATCGCCGTACACGAGGCCGATC
51	V235Q238L239 F	CACGGGTTCGACGCCGRYCGGCAAGMWAVTTGCCTCGCTGGCGGGCCTG
52	V235Q238L239 R	CAGGCCCGCCAGCGAGGCAABTWKCTTGCCGRYCGGCGTCGAACCCGTG
53	R334A337A F	GCTCGCGAACCCGCGCCRSCTGACCGCGATGGCGTCGGTCATCGAC
54	R334A337A R	GTCGATGACCGACGCCATCGCGGTCAGSYGGCGCGGGTTCGCGAGC
55	R334A337K F	GCTCGCGAACCCGCGCCRSCTGACCAAAATGGCGTCGGTCATCGAC
56	R334A337K R	GTCGATGACCGACGCCATTTTGGTCAGSYGGCGCGGGTTCGCGAGC
57	R334A337R F	GCTCGCGAACCCGCGCCRSCTGACCCGTATGGCGTCGGTCATCGAC
58	R334A337R R	GTCGATGACCGACGCCATACGGGTCAGSYGGCGCGGGTTCGCGAGC
59	R334Q/A337R F	GCTCGCGAACCCGCGCCAGCTGACCCGTATGGCGTCGGTCATCGAC
60	R334Q/A337R R	GTCGATGACCGACGCCATACGGGTCAGCTGGCGCGGGTTCGCGAGC

1 Chu, H. S. *et al.* Metabolic engineering of 3-hydroxypropionic acid biosynthesis in Escherichia coli. *Biotechnol Bioeng* **112**, 356-364, doi:10.1002/bit.25444 (2015).

2 Hart, G. J. & Dickinson, F. M. Kinetic properties of highly purified preaparations of sheep liver cytoplasmic aldehyde dehydrogenase. *Biochem. J.* **203**, 617-627, doi:10.1042/bj2030617 (1982).

3 Leicht, W., Heinz, F. & Freimuller, B. Purification and Characterization of Aldehyde Dehydrogepase from bovine liver. *Eur. J. Biochem.* **83**, 189-196, doi:10.1111/j.1432-1033.1978.tb12083.x (1978).

4 Rodriguez-Zavala, J. S. & Weiner, H. Structural Aspects of Aldehyde Dehydrogenase that Influence Dimer-Tetramer Formation. *Biochemistry* **41**, 8229-8237, doi:10.1021/bi012081x (2002).

5 Wierzchowski, J., Pietrzak, M., Szelag, M. & Wroczynski, P. Salivary aldehyde dehydrogenase-reversible oxidation of the enzyme and its inhibition by caffeine, investigated using fluorimetric method. *Arch Oral Biol* **53**, 423-428, doi:10.1016/j.archoralbio.2007.11.004 (2008).

6 Stagos, D. *et al.* Aldehyde dehydrogenase 1B1: molecular cloning and characterization of a novel mitochondrial acetaldehyde-metabolizing enzyme. *Drug Metab Dispos* **38**, 1679-1687, doi:10.1124/dmd.110.034678 (2010).

7 Larson, H. N., Weiner, H. & Hurley, T. D. Disruption of the coenzyme binding site and dimer interface revealed in the crystal structure of mitochondrial aldehyde dehydrogenase "Asian" variant. *J Biol Chem* **280**, 30550-30556, doi:10.1074/jbc.M502345200 (2005).

8 Ho, K. K., Hurley, T. D. & Weiner, H. Selective Alteration of the Rate-Limiting Step in Cytosolic Aldehyde Dehydrogenase through Random Mutagenesis. *Biochemistry* **45**, 9445-9453, doi:10.1021/bi060718c (2006).

9 Ni, L., Zhou, J., Hurley, T. D. & Weiner, H. Human liver mitochondrial aldehyde dehydrogenase Three-dimensional structure and the restoration of solubility and activity of chimeric forms. *Protein Sci* **8**, 2784-2790, doi:10.1110/ps.8.12.2784 (1999).

10 Diaz-Sanchez, A. G. *et al.* Amino acid residues critical for the specificity for betaine aldehyde of the plant ALDH10 isoenzyme involved in the synthesis of glycine betaine. *Plant Physiol* **158**, 1570-1582, doi:10.1104/pp.112.194514 (2012).

11 Rodriguez-Zavala, J. S., Allali-Hassani, A. & Weiner, H. Characterization of E. coli tetrameric aldehyde dehydrogenases with atypical properties compared to other aldehyde dehydrogenases. *Protein Sci* **15**, 1387-1396, doi:10.1110/ps.052039606 (2006).

12 Lindahl, R. & Evces, S. Rat liver aldehyde dehydrogenase. II. Isolation and characterization of four inducible isozymes. *The journal of Biological Chemistry* **259**, 11991-11996 (1984).

13 Vandecasteele, J. P. & Guerrillot, L. [81] Aldehyde dehydrogenases from Pseudomonas aeruginosa. *Methods Enzymol* **89** 484-490, doi:10.1016/s0076-6879(82)89083-6 (1982).

14 Koncitikova, R. *et al.* Role and structural characterization of plant aldehyde

dehydrogenases from family 2 and family 7. *Biochem J* **468**, 109-123, doi:10.1042/BJ20150009 (2015).
